# Simultaneous bilateral fallopian tubal pregnancy of a laparoscopic definite spontaneous unilateral ovulation: a case report

**DOI:** 10.1186/s12884-022-04453-0

**Published:** 2022-02-10

**Authors:** Zhen-zhen Wang, Lian-wei Xu, Li Zhao, Yi-jia Chen, Hui-cong Liu, Er-kai Yu

**Affiliations:** Department of Gynaecology, Longhua Hospital, Shanghai University of Traditional Chinese Medicine, Shanghai, 200032 China

**Keywords:** Bilateral fallopian tubal pregnancy, Laparoscopy, Unilateral ovulation, Case report

## Abstract

**Background:**

Bilateral simultaneous fallopian tubal pregnancy is one of the rarest forms of ectopic pregnancy. Due to the lack of unique features and clinical presentation to distinguish bilateral from unilateral ectopic pregnancy, challenges the diagnosis.

**Case report:**

A 27-year-old Asian woman presented with pelvic pain and vaginal bleeding. Pelvic transvaginal ultrasound showed fluid in Douglas Pouch and posterior fornix puncture revealed unclotted blood. Laparoscopic examination unveiled bilateral ectopic pregnancy with two corpus luteum visible in the right ovary, suggesting a double spontaneous unilateral ovulation. Bilateral fallopian tube fenestration and embryo extraction were performed to preserve fertility.

**Conclusion:**

Diagnosis of bilateral tubal pregnancy is difficult during preoperative ultrasound examination and careful examination during laparoscopic inspection of the whole pelvic cavity to avoid missed diagnosis.

## Background

Ectopic pregnancy, one of the most frequently encountered acute abdominal diseases in gynecology and obstetrics, has an incidence of 1% and a growing prevalence [1]. It is the major cause of maternal death, particularly during the first trimester [1]. The fallopian tube ectopic pregnancy is the most common type and accounts for 90–95% of all ectopic pregnancies [2]. While unilateral tubal ectopic pregnancy is commonly seen, bilateral simultaneous fallopian tubal pregnancy is rare with a reported incidence of 5 in 1 million deliveries and showed a higher incidence among patients with a history of Assisted Reproductive Techniques (ARTs) utilization or following ovulation induction [1, 3, 4]. We herein present an unusual case presented spontaneous simultaneous pregnancy in both fallopian tubes without a history of ARTs. Dual ovulation of the unilateral ovary was evident under direct laparoscopic observation. This article presents some information that should be useful for the clinician, and a further discussion of diagnosis and management on this rare entity is necessary.

## Case presentation

A 27-year-old Asian female presented to our institute with a chief complaint of amenorrhoea for 37 days and vaginal spotting for two days. The patient had a regular menstrual cycle and no history of pelvic surgery, received any infertility treatment, any form of contraception or pelvic inflammatory disease (PID).

Upon presentation, her vitals were regular, with a regular heart rate of 77 beats/ minutes and blood pressure of 120/84 mmHg. Gynecological examination showed no abnormalities besides a small vaginal hemorrhage and tenderness of the bilateral adnexal area. Laboratory examination revealed that blood beta human chorionic gonadotropin (β-hCG) of 2974.00 mIU/mL. The pelvic transvaginal ultrasound examination showed a normal uterus (56 mm*43 mm*52 mm) at the anterior position, an endometrium of 10 mm thick with no gestational sac observed, and an ununiformed posterior uterus wall. Fullness in the Douglas Pouch with an anechoic area of 35 mm*13 mm and a right and left ovary size of 33 mm*25 mm and 30 mm*20 mm, respectively, were observed. A provisional diagnosis of “ectopic pregnancy” was made in our outpatient clinic. Considering the patient has fertility requirements, the patient was admitted for observation of dynamic blood β-hCG and further investigations.

Upon admission in the afternoon, serological examinations, including dynamic blood β-hCG, were requested, and the patient was scheduled for surgical intervention the next day. The following day, serological studies showed a blood β-hCG 3091.00 mIU/mL, progesterone 36.5 nmol/L, and no significant increase in blood β-hCG compared to the outpatient clinic result. The combination of the symptoms, blood β-hCG titer, and pelvic transvaginal ultrasound examination results support the standard diagnosis of “ectopic pregnancy.” Furthermore, the patient compliant of dull pain in the lower abdomen, and the re-examination pelvic ultrasound revealed that the intimal thickness of 13 mm, a hypoechoic heterogeneity at a size of 22–24 in the left pelvis with a clear boundary and peripheral blood flow. Fluid in Douglas Pouch(48*23 mm) and posterior fornix puncture revealed 2 mL of unclotted blood. Considering the complications of ectopic pregnancy includes abdominal bleeding and hemorrhagic shock, the laparoscopic operation was performed immediately with the consent of the patient and her family.

**Fig. 1:**
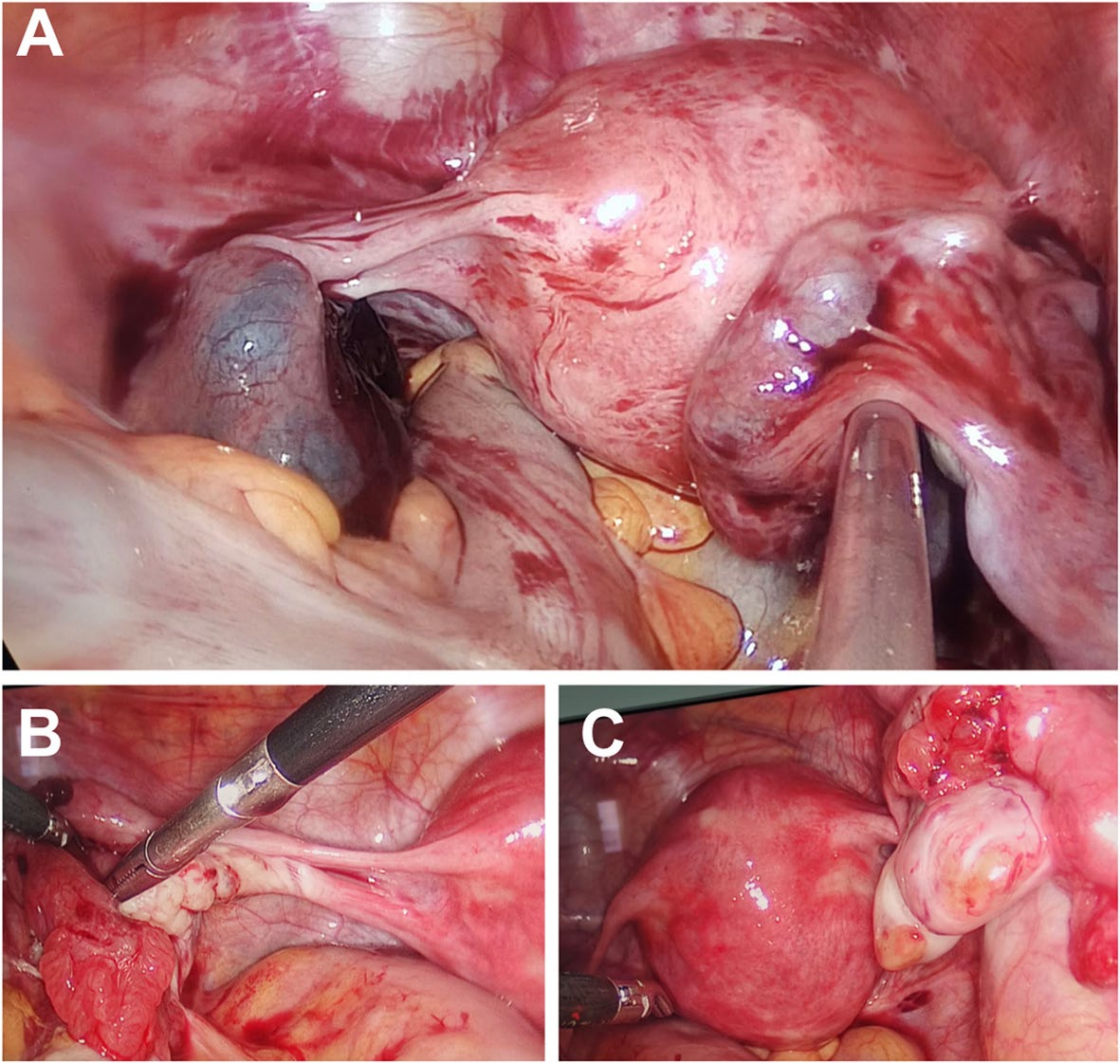
Intraoperatively laparoscopic examination **A** revealed a bilateral fallopian tube pregnancy, **B** no corpus luteum was visualized in the left ovary, and **C** two corpus luteum were visualized in the right ovary after ovulation

**Fig. 2:**
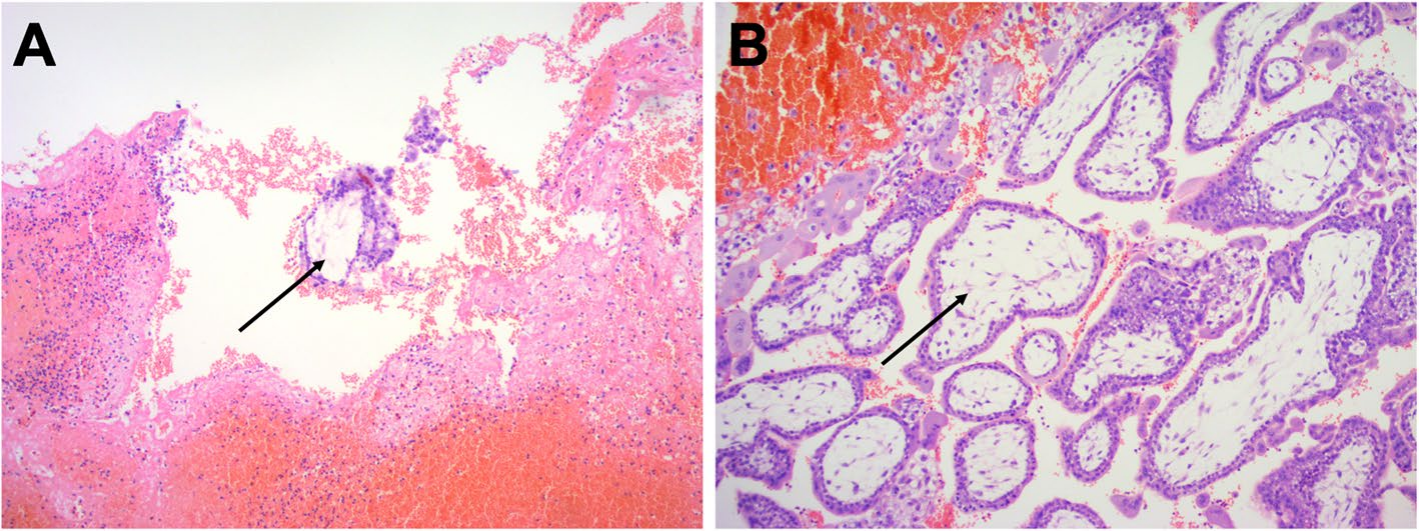
Pathological examination of **A** the blood clot and the contents of the right fallopian tube, **B** the blood clot and the contents of the left fallopian tube, and the black arrows indicate the chorionic villi tissues

Intra-operatively, an enlarged and intact ampulla of the left fallopian tube (4*3*3 cm) with hemorrhage blocks at the umbrella end was observed. The ampulla of the right fallopian tube was enlarged (3*2*2 cm). In contrast, the right ovary was normal in size, but with two corpus luteum, suggesting a double spontaneous unilateral ovulation (Fig. 1). No abnormality was found in the left ovary. Bilateral fallopian tube fenestration and embryo extraction were performed following the consent of the family members with signatures. Following the embryo removal inside of bilateral fallopian tubes, hematocele and chorionic villi tissues were observed after dissection. Bilateral fallopian tube mesangium and fallopian tube fenestrated were injected with 50 mg Methotrexate (MTX) to prevent pregnancy. Pathological examination results showed the presence of chorionic villi tissues in the blood clot and the contents of both the left and the right fallopian tubes (Fig. 2). Postoperatively, nutritional support treatment was provided to promote recovery. Serological examination showed a blood β-hCG of 768.99 mIU/mL and 254.61 mIU/mL two and four days postoperatively. The patient’s vitals were stable, and she was discharged. The patient was subjected to follow-up for one month on outpatient clinics until blood β-hCG was normal.

## Discussion and conclusion

Ectopic pregnancy is a prevalent gynecological disease commonly caused by anatomically abnormal fallopian tubes, migration of fertilized eggs, failure of contraception, and implementation of assisted reproductive technology [5]. According to Hellin-Zeleny’s law, the formula for calculating the probability of multiple gestations in a natural state is 1/89^n−1^, where *n* is the number of fetuses in a singular pregnancy. Based on the formula, the probability of twins in a natural state is approximately 1%; among them, dizygotic twins account for about 70% of pregnancy, and monozygotic twins account for about 30% of pregnancy. Although natural twins are caused by either unilateral or bilateral ovulation, and no statistical analysis has been currently reported. Simultaneous pregnancy of bilateral fallopian tubes is rare, with an incidence of 0.03% [6], and commonly occurs among those with a history of assisted ARTs or multiple birth genes in the family.

In the present case, the patient had no history of ARTs assisted reproduction, no evidence of dysmorphic uterus, and no family history. The patient developed a spontaneous bilateral tubal pregnancy and no signs of ovulation in the left ovary under laparoscopy. Two corpus luteum were visualized in the right ovary after ovulation. To our knowledge, it is particularly rare to encounter ectopic pregnancy caused by the successful discharge of two eggs from the right ovary and delivered to each side of the fallopian tube. If the chorionic villi tissues of bilateral ectopic pregnancy could be genetically detected, it would be more accurate to determine whether the present pregnancy was dizygotic or monozygotic twins.

It is worth noting that the diagnosis of bilateral tubal pregnancy was difficult to be established. First, it was difficult to establish a diagnosis during the preoperative ultrasound examination. Second, when performing the laparoscopic examination, particular attention is needed to inspect the whole pelvic cavity in detail. In the present case, the laparoscopic observation of left tubal pregnancy was easily visualized, while the pregnancy on the right side of the fallopian tube was more concealed, where there was only a slight bulge visualized at the ampulla, as the umbrella end without a tear or blood clots can be easily neglected upon inspection. Incomplete surgery could also lead to a missed diagnosis and the risk of repeated operation. In this case, the simultaneous bilateral fallopian tube pregnancy occurred with intact fallopian tubes, and the patient is fertile. Therefore, fenestration and embryo extraction was performed to preserve the integrity of the organs. Local injection of methotrexate was given postoperatively to prevent residual chorionic villi tissues. The blood β-hCG level of postoperative follow-up decreased markedly, which was a successful case with a reference value. To conclude, this successful management of bilateral tubal pregnancy shows that a careful inspection of both tubes, even in the absence of tear or blood clots at the umbrella end, is needed during laparoscopy examination and/or operation is needed to avoid missing this rare life-threatening condition.

## Data Availability

Data available upon written request addressed to the corresponding author.

## Abbreviations

ARTs: Assisted Reproductive Techniques
PID: Pelvic Inflammatory Disease
hCG: Human Chorionic Gonadotropin
MTX: Methotrexate
